# Supplementary material for: A comparative study on the use of microscopy in pharmacology and cell biology research
Source: PLoS One. 2021 Jan 22;16(1):e0245795. doi: 10.1371/journal.pone.0245795 (PMC7822289; doi:10.1371/journal.pone.0245795)
Supplement: S1 File — The techniques analyzed were bright field (BrF), phase contrast (Pha), differential interference contrast (DIC), polarization (Pol), conventional fluorescence (Flu), confocal fluorescence (Conf), super resolution (SRes), and live cell imaging (Live); and 3 types of electron microscopy techniques: transmission (TEM), scanning (SEM) and cryo-EM (CrEM). (DOCX) [file pone.0245795.s001.docx]

| **# of combined**  **techniques** | **Microscopy techniques** | **# of articles** |
| --- | --- | --- |
| 6 | BrF DIC Flu Conf Live SEM | 1 |
| 6 | BrF Flu Conf SRes Live TEM | 1 |
| 5 | BrF Pha Flu Conf Live | 3 |
| 5 | BrF Flu Conf Live TEM | 2 |
| 5 | DIC Flu Conf Live TEM | 2 |
| 5 | BrF DIC Conf SRes TEM | 1 |
| 5 | BrF DIC Flu Conf Live | 1 |
| 5 | BrF Flu Conf Live SEM | 1 |
| 5 | BrF Flu Conf SEM TEM | 1 |
| 5 | BrF Flu Conf SRes Live | 1 |
| 5 | BrF Pha DIC Flu SEM | 1 |
| 5 | Conf SRes Live SEM TEM | 1 |
| 5 | DIC Pol Conf Live TEM | 1 |
| 5 | Flu Conf SRes Live TEM | 1 |
| 5 | Pha DIC Conf Live TEM | 1 |
| 5 | Pha DIC Flu Live TEM | 1 |
| 5 | Pha Flu Conf SRes Live | 1 |
| 4 | DIC Flu Conf Live | 6 |
| 4 | BrF Flu Conf Live | 4 |
| 4 | BrF Pha Flu Conf | 4 |
| 4 | Flu Conf SRes Live | 3 |
| 4 | BrF Flu Conf SEM | 2 |
| 4 | BrF Flu Conf TEM | 2 |
| 4 | BrF Pha Conf TEM | 2 |
| 4 | BrF Pha DIC Flu | 2 |
| 4 | BrF Pha Flu TEM | 2 |
| 4 | DIC Conf SRes Live | 2 |
| 4 | DIC Flu Live TEM | 2 |
| 4 | Flu Conf SRes TEM | 2 |
| 4 | Pha Conf Live SEM | 2 |
| 4 | Pha Flu Conf Live | 2 |
| 4 | Pha Flu Conf TEM | 2 |
| 4 | Pha Flu Live TEM | 2 |
| 4 | BrF Conf CrEM SEM | 1 |
| 4 | BrF Conf SEM TEM | 1 |
| 4 | BrF DIC Conf Live | 1 |
| 4 | BrF Flu Live TEM | 1 |
| 4 | BrF Pha DIC Conf | 1 |
| 4 | BrF Pha Flu TEM | 1 |
| 4 | BrF Pha SRes TEM | 1 |
| 4 | BrF SRes Live TEM | 1 |
| 4 | Conf Live SEM TEM | 1 |
| 4 | Conf SRes Live SEM | 1 |
| 4 | DIC Flu Conf SEM | 1 |
| 4 | DIC Flu Conf TEM | 1 |
| 4 | DIC Flu CrEM TEM | 1 |
| 4 | DIC Flu SRes Live | 1 |
| 4 | DIC Flu SRes SEM | 1 |
| 4 | DIC Flu SRes TEM | 1 |
| 4 | Flu Conf Live SEM | 1 |
| 4 | Flu Conf Live TEM | 1 |
| 4 | Flu Conf SEM TEM | 1 |
| 4 | Pha Conf SRes Live | 1 |
| 4 | Pha DIC Flu Conf | 1 |
| 3 | BrF Flu Conf | 21 |
| 3 | BrF Pha Flu | 18 |
| 3 | Flu Conf Live | 18 |
| 3 | Conf SRes Live | 7 |
| 3 | Pha Flu Conf | 7 |
| 3 | BrF Conf Live | 6 |
| 3 | BrF Conf TEM | 6 |
| 3 | BrF Flu Live | 6 |
| 3 | BrF Flu TEM | 6 |
| 3 | Conf Live TEM | 6 |
| 3 | DIC Conf Live | 6 |
| 3 | Flu Conf TEM | 6 |
| 3 | Pha Conf Live | 6 |
| 3 | DIC Flu Conf | 5 |
| 3 | BrF Pha Conf | 4 |
| 3 | Conf SRes TEM | 4 |
| 3 | DIC Flu TEM | 4 |
| 3 | BrF Flu SEM | 3 |
| 3 | Flu Conf SRes | 3 |
| 3 | Flu SRes Live | 3 |
| 3 | Pha Flu Live | 3 |
| 3 | BrF DIC Flu | 2 |
| 3 | Conf SEM TEM | 2 |
| 3 | DIC Conf TEM | 2 |
| 3 | Pha DIC Flu | 2 |
| 3 | BrF DIC Conf | 1 |
| 3 | BrF Live SEM | 1 |
| 3 | BrF Pha Live | 1 |
| 3 | BrF Pha SEM | 1 |
| 3 | BrF Pha TEM | 1 |
| 3 | BrF Pol Conf | 1 |
| 3 | BrF Pol SEM | 1 |
| 3 | DIC Flu Live | 1 |
| 3 | Flu Conf SEM | 1 |
| 3 | Flu CrEM TEM | 1 |
| 3 | Flu Live TEM | 1 |
| 3 | Pha Conf SEM | 1 |
| 3 | Pha CrEM SEM | 1 |
| 3 | Pha CrEM TEM | 1 |
| 3 | Pha Flu SEM | 1 |
| 3 | Pha Flu SRes | 1 |
| 2 | BrF Flu | 97 |
| 2 | BrF Conf | 53 |
| 2 | Flu Conf | 42 |
| 2 | Conf Live | 41 |
| 2 | Pha Flu | 25 |
| 2 | Conf TEM | 22 |
| 2 | Flu Live | 16 |
| 2 | Flu TEM | 14 |
| 2 | Pha Conf | 12 |
| 2 | BrF Pha | 11 |
| 2 | BrF TEM | 8 |
| 2 | DIC Flu | 7 |
| 2 | DIC Flu | 6 |
| 2 | BrF SEM | 4 |
| 2 | Flu SRes | 4 |
| 2 | SEM TEM | 4 |
| 2 | DIC Conf | 3 |
| 2 | Flu CrEM | 3 |
| 2 | Conf SRes | 2 |
| 2 | CrEM TEM | 2 |
| 2 | Flu SEM | 2 |
| 2 | Conf SEM | 1 |
| 2 | CrEM Live | 1 |
| 2 | DIC Live | 1 |
| 2 | Pha SEM | 1 |
| 2 | Pha TEM | 1 |
| 2 | SRes Live | 1 |
| 2 | SRes TEM | 1 |

**S1 File -** Frequency of the combinations of microscopy techniques used simultaneously in the articles from eight biomedical sciences journals. The techniques analyzed were bright field (BrF), phase contrast (Pha), differential interference contrast (DIC), polarization (Pol), conventional fluorescence (Flu), confocal fluorescence (Conf), super resolution (SRes), and live cell imaging (Live); and 3 types of electron microscopy techniques: transmission (TEM), scanning (SEM) and cryo-EM (CrEM).
