# Supplementary material for: A comparative study on the use of microscopy in pharmacology and cell biology research
Source: PLoS One. 2021 Jan 22;16(1):e0245795. doi: 10.1371/journal.pone.0245795 (PMC7822289; doi:10.1371/journal.pone.0245795)
Supplement: S2 File — British Journal of Pharmacology (BJP), Journal of Pharmacy and Pharmacology (JPP), Frontiers in Pharmacology (FP), Journal of Cell Biology (JCB), Journal of Cell Science (JCS), Cells (CEL), Journal of Biological Chemistry (JBC) and Proceedings of the National Academy of Sciences (PNAS). The numbers at the left column of each journal refers to the number of times that each word appeared in the title of the articles (N = 200) from this journal in 2019. (DOCX) [file pone.0245795.s002.docx]

|  | **BJP** |  | **FP** |  | **JPP** |  | **JCB** |  | **JCS** |  | **CEL** |  | **JBC** |  | **PNAS** |
| --- | --- | --- | --- | --- | --- | --- | --- | --- | --- | --- | --- | --- | --- | --- | --- |
| 34 | receptor | 34 | inhibitior | 40 | cell | 35 | regulation | 50 | cell | 94 | cell | 54 | protein | 23 | cell |
| 29 | inhibitor | 29 | cell | 28 | inhibitor | 36 | cell | 33 | regulation | 24 | cancer | 26 | cell | 12 | activity |
| 21 | cell | 20 | cancer | 23 | rat | 23 | protein | 22 | protein | 22 | human | 19 | complex | 12 | protein |
| 21 | mouse | 15 | pathway | 20 | cancer | 18 | control | 17 | signalling | 20 | model | 15 | human | 11 | human |
| 19 | channel | 14 | signalling | 17 | vitro | 13 | signalling | 12 | complex | 19 | expression | 14 | kinase | 10 | structure |
| 17 | rat | 11 | attenuate | 16 | drug | 12 | microtubule | 11 | membrane | 19 | inhibitor | 13 | receptor | 8 | cancer |
| 14 | signalling | 11 | inflammation | 15 | activity | 9 | assembly | 10 | actin | 16 | effect | 12 | factor | 7 | dna |
| 12 | human | 11 | patient | 15 | injury | 9 | droplet | 10 | activation | 16 | signalling | 12 | mouse | 7 | dynamic |
| 11 | attenuate | 10 | suppress | 15 | pathway | 9 | growth | 10 | assembly | 14 | mouse | 11 | expression | 6 | interaction |
| 11 | disease | 9 | stress | 14 | mouse | 9 | nuclear | 10 | localization | 14 | stem | 10 | inhibitor | 6 | receptor |
| 11 | activity | 8 | acute | 13 | human | 8 | activation | 9 | drosophila | 13 | mesenchymal | 10 | regulate | 6 | reveal |
| 11 | induce | 8 | apoptosis | 13 | mediate | 8 | chromosome | 9 | function | 13 | novel | 10 | ubiquitin | 5 | deficiency |
| 10 | inflammation | 8 | expression | 15 | model | 8 | membrane | 9 | nuclear | 11 | activation | 9 | activity | 5 | factor |
| 10 | pathway | 7 | breast | 11 | signalling | 8 | transport | 9 | pathway | 11 | pathway | 9 | ligase | 5 | regulate |
| 9 | hypertension | 7 | herbal | 10 | apoptosis | 7 | actin | 9 | yeast | 10 | extracellular | 8 | dna | 5 | response |
| 8 | agonist | 7 | human | 10 | extract | 7 | complex | 8 | calcium | 10 | growth | 8 | gene | 5 | signalling |
| 8 | vascular | 7 | injury | 10 | potential | 7 | kinetochore | 8 | differentiation | 10 | liver | 8 | transcription | 5 | stem |
| 7 | dysfunction | 7 | lung | 9 | factor | 7 | lipid | 8 | integrin | 13 | receptor | 7 | binding | 5 | tumor |
| 7 | fibrosis | 7 | mechanism | 9 | inflammatory | 7 | polarity | 8 | interaction | 9 | tumor | 7 | functional | 4 | antibody |
| 7 | injury | 7 | molecular | 9 | pharmacokinetics | 6 | development | 8 | receptor | 8 | disease | 7 | mitochondrial | 4 | complex |
| 7 | muscle | 6 | activity | 9 | stress | 6 | dynamic | 7 | dynamic | 8 | epithelial | 7 | promote | 4 | disease |
| 7 | protein | 6 | formula | 8 | attenuate | 6 | migration | 7 | migration | 8 | induce | 7 | synthase | 4 | immune |
| 7 | target | 6 | function | 8 | receptor | 6 | mitochondrial | 7 | mouse | 7 | apoptosis | 7 | yeast | 4 | pathway |
| 7 | vivo | 6 | mouse | 7 | characterization | 6 | mitotic | 6 | expression | 7 | carcinoma | 6 | breast | 4 | potential |
| 7 | model | 6 | profile | 7 | combination | 6 | recruitment | 6 | induces | 7 | macrophage | 6 | cancer | 4 | stimulation |
| 6 | alleviate | 6 | protective | 7 | delivery | 5 | apical | 6 | microtubule | 7 | mitochondrial | 6 | channel | 10 | target |
| 6 | binding | 6 | protein | 7 | evaluation | 5 | aurora | 6 | mitochondrial | 7 | muscle | 6 | control | 3 | active |
| 6 | cancer | 6 | response | 7 | loaded | 5 | autophagosome | 6 | muscle | 7 | transition | 6 | domain | 3 | adaptive |
| 6 | kinase | 6 | syndrome | 7 | oxidative | 5 | lysosome | 5 | activity | 7 | vitro | 9 | mechanism | 3 | bacteria |
| 6 | liver | 6 | therapeutic | 7 | protect | 5 | mediate | 5 | cancer | 6 | activity | 6 | mediate | 3 | brain |
| 6 | mechanism | 6 | treatment | 7 | resistance | 5 | release | 5 | elegans | 6 | factor | 6 | phosphorylation | 3 | breast |
| 6 | mouse | 5 | activation | 7 | treatment | 5 | spindle | 5 | golgi | 6 | metabolic | 6 | signalling | 3 | coli |
| 6 | protect | 5 | ameliorate | 6 | autophagy | 4 | active | 5 | homeostasis | 6 | protein | 6 | site | 3 | cycle |
| 6 | transporter | 5 | development | 6 | brain | 4 | axon | 5 | invasion | 6 | response | 6 | stress | 3 | function |
| 6 | neuron | 5 | disease | 6 | kidney | 4 | binding | 5 | kinase | 6 | stress | 6 | structure | 3 | gene |
| 6 | therapy | 5 | growth | 6 | liver | 4 | cancer | 5 | modulate | 6 | vesicle | 5 | activation | 3 | growth |
| 5 | ameliorate | 5 | injection | 6 | lung | 4 | cargo | 5 | phosphorylation | 6 | virus | 5 | coli | 3 | host |
| 5 | angiotensin | 5 | metabolic | 6 | renal | 4 | checkpoint | 5 | RNA | 6 | vivo | 5 | drug | 6 | human |
| 5 | apoptosis | 5 | novel | 6 | system | 4 | cytoplasmic | 5 | skeletal | 5 | 3D | 5 | escherichia | 3 | imaging |
| 5 | cardiac | 5 | oxidative | 6 | vivo | 4 | domain | 5 | spindle | 5 | brain | 5 | provide | 3 | infection |
| 5 | cerebral | 5 | pharmacokinetics | 5 | acute | 4 | dynein | 5 | trafficking | 5 | development | 5 | recognition | 3 | inhibition |
| 5 | diabetic | 5 | prevent | 5 | breast | 4 | epithelial | 5 | transport | 5 | differentiation | 9 | regulate | 3 | mechanism |
| 5 | endothelial | 5 | proliferation | 5 | cytotoxicity | 4 | focal | 4 | fission | 5 | dna | 5 | ribosomal | 3 | metabolism |
| 5 | experimental | 4 | action | 5 | disease | 4 | formation | 4 | growth | 5 | hepatic | 4 | active | 3 | model |
| 5 | expression | 4 | cardiac | 5 | glycoprotein | 4 | fusion | 4 | mitotic | 5 | inflammation | 4 | assembly | 3 | plant |
| 5 | factor | 4 | combination | 5 | intestinal | 4 | gtpase | 4 | plasma | 5 | injury | 4 | bacteria | 3 | plasma |
| 5 | hepatic | 4 | component | 5 | mechanism | 4 | kinase | 4 | proliferation | 5 | phenotype | 4 | chaperone | 3 | prostate |
| 5 | intestinal | 4 | decoction | 5 | oral | 4 | ligase | 4 | tumor | 5 | rat | 4 | degradation | 3 | regulatory |
| 5 | modulate | 4 | downregulation | 9 | regulation | 4 | meiosis | 3 | apical | 5 | regulation | 4 | interaction | 3 | requires |
| 4 | atp | 4 | factor | 5 | specie | 4 | modulate | 3 | axon | 5 | stromal | 4 | liver | 3 | simulation |
| 4 | brain | 4 | fibrosis | 4 | absorption | 4 | morphogenesis | 3 | biogenesis | 5 | tissue | 4 | molecular | 3 | site |
| 4 | functional | 4 | inflammatory | 4 | apoptotic | 4 | MT1-MMP | 3 | budding | 5 | transcriptional | 4 | protease | 3 | stability |
| 4 | mitochondria | 4 | integrated | 4 | behaviour | 4 | phosphorylation | 3 | channel | 5 | upregulation | 4 | remodeling | 3 | suppress |
| 4 | nicotinic | 4 | kinase | 4 | bioavailability | 4 | secretion | 3 | chromatin | 4 | angiogenesis | 4 | site | 3 | surface |
| 4 | pain | 4 | liver | 4 | cardiac | 4 | segregation | 3 | cilia | 4 | arthritis | 4 | structural | 3 | synthesis |
| 4 | phosphorylation | 4 | mechanism | 4 | chronic | 4 | stabilize | 3 | cycle | 4 | breast | 4 | transmembrane | 3 | transcription |
| 4 | pulmonary | 4 | metabolomic | 4 | determination | 4 | tissue | 3 | cytokinesis | 4 | cardiac | 4 | transporter | 3 | vivo |
| 4 | regulation | 4 | natural | 4 | development | 3 | activity | 3 | degradation | 4 | death | 3 | acetylation |  |  |
| 4 | release | 4 | potential | 4 | diabetic | 3 | adaptor | 3 | dendritic | 4 | deficiency | 3 | acyltransferase |  |  |
| 4 | response | 4 | target | 4 | dose | 6 | adhesion | 3 | endocytic | 4 | endothelial | 3 | atp |  |  |
| 4 | smooth | 4 | trial | 4 | endoplasmic | 3 | anaphase | 3 | envelope | 4 | neutrophil | 3 | autophagy |  |  |
| 4 | sulfide | 3 | acetaminophen-Induced | 4 | enzyme | 3 | barrier | 3 | erk1/2 | 4 | ovarian | 3 | brain |  |  |
| 4 | toll-like | 3 | active | 4 | expression | 3 | biogenesis | 3 | fatty | 4 | oxidative | 3 | catalytic |  |  |
| 3 | action | 3 | affects | 4 | extract | 3 | contact | 3 | flagellar | 4 | pancreatic | 3 | cleavage |  |  |
| 3 | acute | 3 | airway | 4 | fatty | 3 | cooperate | 3 | histone | 4 | primary | 3 | crystal |  |  |
| 3 | adenosine | 3 | akt | 4 | fibrosis | 3 | cycle | 3 | inhibition | 4 | transplantation | 3 | damage |  |  |
| 3 | allosteric | 3 | angiotensin | 4 | hypericum | 3 | dependent | 3 | integrity | 3 | adhesion | 3 | death |  |  |
| 3 | aortic | 3 | atrial | 4 | immune | 3 | determine | 3 | intracellular | 3 | adipose | 3 | differentiation |  |  |
| 3 | arterial | 3 | capsule | 4 | inflammation | 3 | dna | 3 | macropinocytosis | 3 | biomarkers | 3 | disease |  |  |
| 3 | cardiomyocyte | 3 | carbon | 4 | lipid | 3 | endocytosis | 3 | mammalian | 3 | cell-derived | 3 | extracellular |  |  |
| 3 | epithelial | 3 | carcinoma | 4 | miR | 3 | human | 3 | mediate | 3 | culture | 3 | factor |  |  |
| 3 | fatty | 3 | clinical | 4 | nanoparticle | 3 | imaging | 3 | membrane | 3 | cycle | 3 | gene |  |  |
| 3 | function | 3 | colorectal | 4 | pain | 3 | inner | 3 | mitochondria | 3 | dynamics | 3 | heme |  |  |
| 3 | gene | 3 | derivatives | 4 | pathway | 3 | interaction | 3 | mitosis | 3 | engineered | 3 | high-throughput |  |  |
| 3 | inflammatory | 3 | diabetes | 4 | pharmacokinetic | 3 | intercellular | 3 | mutation | 3 | fat | 3 | homology |  |  |
| 3 | insulin | 3 | diet | 4 | preparation | 3 | intracellular | 3 | network | 3 | fibrosis | 3 | insulin |  |  |
| 3 | metabolic | 3 | differentiation | 4 | proliferation | 3 | intrinsic | 3 | neuron | 3 | functional | 3 | ligand |  |  |
| 3 | morphine | 3 | drug | 4 | protein | 3 | junction | 3 | nuclear | 3 | genetic | 3 | lipid |  |  |
| 3 | opioid | 3 | dysfunction | 4 | rat | 3 | localization | 3 | phosphorylation | 3 | homeostasis | 3 | membrane |  |  |
| 3 | oxidative | 3 | endoplasmic | 4 | release | 3 | mediated | 3 | polarity | 3 | host | 3 | mucin |  |  |
| 3 | peptide | 3 | extract | 4 | response | 3 | mitosis | 3 | primary | 3 | hypoxia | 3 | myosin |  |  |
| 3 | pharmacological | 3 | factor | 4 | role | 3 | motility | 3 | release | 3 | induce | 3 | neuron |  |  |
| 3 | potent | 3 | gene | 4 | serum | 3 | myosin | 3 | require | 3 | infection | 3 | nuclear |  |  |
| 3 | potential | 3 | ginsenoside | 4 | stem | 6 | neuron | 3 | response | 3 | inhibit | 3 | pancreatic |  |  |
| 3 | renal | 3 | gut | 4 | targeting | 3 | organization | 3 | segregation | 3 | iron | 3 | phosphatase |  |  |
| 3 | selective | 3 | heart | 3 | activation | 3 | p53 | 3 | signalling | 3 | long-term | 3 | polymerase |  |  |
| 3 | survival | 3 | hepatotoxicity | 3 | administration | 3 | pathway | 3 | suppress | 3 | metabolism | 3 | pump |  |  |
| 3 | syndrome | 3 | hepg2 | 3 | ameliorate | 3 | progression | 3 | target | 3 | metastatic | 3 | regulation |  |  |
| 3 | tissue | 3 | high-Fat | 3 | antioxidant | 3 | region | 3 | tau | 3 | migration | 3 | resistance |  |  |
| 3 | up-regulate | 3 | iron | 3 | binding | 3 | regulate | 6 | transcription | 3 | miRNA | 3 | rna |  |  |
| 3 | vasodilation | 3 | kidney | 3 | bone | 3 | remodeling | 3 | variant | 3 | modulate | 3 | secretion |  |  |
|  |  | 3 | metastasis | 3 | caco | 3 | scaffold |  |  | 3 | molecule | 3 | stimulate |  |  |
|  |  | 3 | migration | 3 | cerebral | 3 | secretory |  |  | 3 | patient | 3 | structural |  |  |
|  |  | 3 | model | 3 | collagen | 3 | sorting |  |  | 3 | pluripotent | 3 | substrate |  |  |
|  |  | 3 | nf-κb | 3 | compound | 3 | stability |  |  | 3 | profile | 3 | subunit |  |  |
|  |  | 3 | patchouli | 3 | crocus | 3 | stress |  |  | 3 | proliferation | 3 | T-cell |  |  |
|  |  | 3 | pharmacological | 3 | culture | 3 | survival |  |  | 3 | regeneration | 3 | transcriptional |  |  |
|  |  | 3 | phosphorylation | 3 | cytochrome | 3 | trafficking |  |  | 6 | regulation | 3 | transport |  |  |
|  |  | 3 | pulmonary | 3 | damage | 3 | transition |  |  | 3 | RNA | 3 | tyrosine |  |  |
|  |  | 3 | randomized | 3 | development | 3 | tumor |  |  | 3 | sclerosis |  |  |  |  |
|  |  | 3 | receptor | 3 | doxorubicin | 3 | zone |  |  | 3 | skeletal |  |  |  |  |
|  |  | 3 | resistance | 3 | efflux |  |  |  |  | 3 | spinal |  |  |  |  |
|  |  | 3 | reticulum | 3 | fruit |  |  |  |  | 3 | stellate |  |  |  |  |
|  |  | 3 | screening | 3 | gastric |  |  |  |  | 3 | stemness |  |  |  |  |
|  |  |  |  | 3 | growth |  |  |  |  | 3 | therapy |  |  |  |  |
|  |  |  |  | 3 | healing |  |  |  |  | 3 | toxicity |  |  |  |  |
|  |  |  |  | 3 | heart |  |  |  |  | 3 | trap |  |  |  |  |
|  |  |  |  | 3 | hydrogel |  |  |  |  | 3 | vascular |  |  |  |  |
|  |  |  |  | 3 | hydrolase |  |  |  |  |  |  |  |  |  |  |
|  |  |  |  | 3 | hypericin |  |  |  |  |  |  |  |  |  |  |
|  |  |  |  | 3 | hypertensive |  |  |  |  |  |  |  |  |  |  |
|  |  |  |  | 3 | induce |  |  |  |  |  |  |  |  |  |  |
|  |  |  |  | 3 | inhibit |  |  |  |  |  |  |  |  |  |  |
|  |  |  |  | 3 | lipopolysaccharide |  |  |  |  |  |  |  |  |  |  |
|  |  |  |  | 3 | local |  |  |  |  |  |  |  |  |  |  |
|  |  |  |  | 3 | metabolism |  |  |  |  |  |  |  |  |  |  |
|  |  |  |  | 3 | nanoemulsion |  |  |  |  |  |  |  |  |  |  |
|  |  |  |  | 3 | neuroinflammation |  |  |  |  |  |  |  |  |  |  |
|  |  |  |  | 3 | neuropathy |  |  |  |  |  |  |  |  |  |  |
|  |  |  |  | 3 | nuclear |  |  |  |  |  |  |  |  |  |  |
|  |  |  |  | 3 | oil |  |  |  |  |  |  |  |  |  |  |
|  |  |  |  | 3 | oxidase |  |  |  |  |  |  |  |  |  |  |
|  |  |  |  | 3 | oxygen |  |  |  |  |  |  |  |  |  |  |
|  |  |  |  | 3 | patient |  |  |  |  |  |  |  |  |  |  |
|  |  |  |  | 3 | peripheral |  |  |  |  |  |  |  |  |  |  |
|  |  |  |  | 3 | protective |  |  |  |  |  |  |  |  |  |  |
|  |  |  |  | 3 | pulmonary |  |  |  |  |  |  |  |  |  |  |
|  |  |  |  | 3 | restore |  |  |  |  |  |  |  |  |  |  |
|  |  |  |  | 3 | resveratrol |  |  |  |  |  |  |  |  |  |  |
|  |  |  |  | 3 | reticulum |  |  |  |  |  |  |  |  |  |  |
|  |  |  |  | 3 | reverse |  |  |  |  |  |  |  |  |  |  |
|  |  |  |  | 3 | risk |  |  |  |  |  |  |  |  |  |  |
|  |  |  |  | 3 | saffron |  |  |  |  |  |  |  |  |  |  |
|  |  |  |  | 3 | sativus |  |  |  |  |  |  |  |  |  |  |
|  |  |  |  | 3 | situ |  |  |  |  |  |  |  |  |  |  |
|  |  |  |  | 3 | solid |  |  |  |  |  |  |  |  |  |  |
|  |  |  |  | 3 | tablet |  |  |  |  |  |  |  |  |  |  |
|  |  |  |  | 3 | transport |  |  |  |  |  |  |  |  |  |  |
|  |  |  |  | 3 | wound |  |  |  |  |  |  |  |  |  |  |

**S2 File -** Frequency of words used in the title of articles from eight biomedical sciences journals. British Journal of Pharmacology (BJP), Journal of Pharmacy and Pharmacology (JPP), Frontiers in Pharmacology (FP), Journal of Cell Biology (JCB), Journal of Cell Science (JCS), Cells (CEL), Journal of Biological Chemistry (JBC) and Proceedings of the National Academy of Sciences (PNAS). The numbers at the left column of each journal refers to the number of times that each word appeared in the title of the articles (N = 200) from this journal in 2019.
