# Supplementary material for: A comparative study on the use of microscopy in pharmacology and cell biology research
Source: PLoS One. 2021 Jan 22;16(1):e0245795. doi: 10.1371/journal.pone.0245795 (PMC7822289; doi:10.1371/journal.pone.0245795)
Supplement: Attachment — Submitted filename: Response to Reviewers.doc [file pone.0245795.s004.doc]

# From: Prof. **Claudia dos Santos Mermelstein**

#	Instituto de Ciências Biomédicas

#	Universidade Federal do Rio de Janeiro

#	Rio de Janeiro, Brazil

e-mail: mermelstein@histo.ufrj.br

To:	**Plos One**

Rio de Janeiro, January 05, 2021.

Dear Dr. **Yuval Garini**,

I submitted the manuscript entitled "**A comparative study on the use of microscopy in pharmacology and cell biology research**" (**PONE-D-20-26569R1**) for publication in PLOS ONE. After careful consideration, it was considered that the manuscript has merit but does not fully meet PLOS ONE’s publication criteria as it currently stands, pending minor revisions. Therefore, I made the modifications in the manuscript and in its figures and I included a point-by-point response to the reviewer’s comments, which were very useful to improve the data presentation and interpretation. New figures were added. I thank the Editor for his careful and appropriate analysis.

**Editor comments**:

The manuscript is now improved according to all the comments and requests by the reviewers. There are still few issues to be refined as listed below.

1 - PNAS was one of the journals that was evaluated. Note however, that PNAS is a general journal that publish articles in many subjects that are outside the scope of cell biology, or even biology. Consider how to take this into account (i.e. when quoting in page 8 the percentage of OM, the value is lower, 32%, but I believe that if this will be normalized by the percentage of bio-related publications from all publications, the number will become more similar to that of the cell-bio journals).

**Author’s response** – We thank the editor for this important comment. We now analyzed the percentage of OM use in bio-related (biological sciences section of the journal) articles from PNAS. In fact, the percentage of OM in all PNAS’s articles was 32%, whereas the percentage of OM only in bio-related articles is 47%. So, we incorporated this new analysis in the new version of the manuscript.

2 - Page 8: “Optical microscopy (OM) was much more used than electron microscopy (EM), probably because EM is more labor-intensive than OM”. There are many reasons for the broader use of optical microscopy rather than EM, except for the labor issue. It is also because it is faster, possible to label multiple probes simultaneously, works on live cells, the structure is not damaged during the preparation, it is fully quantitative, and even though the resolution is not as good, it is still good enough to provide sub-cellular information. Most likely, it is more accurate to claim that EM is used only where very high resolution is needed, and OM cannot provide that resolution.

**Author’s response** – We agree with the editor that there are many reasons for the broader use of optical microscopy rather than EM, except for the labor issue. So, we changed this sentence accordingly.

3 - Page 9: “and an image of the object of study should be almost a requirement for these studies”. It is not clear where is this fact coming from? Please check the argument, and if necessary, please provide a reference.

**Author’s response** – We rephrased this sentence since it was not clear.

4 - Page 10 lines 222: Cryo-EM is most likely being used for basic-science studies, which explains the low-percentage use.

**Author’s response** – We added this sentence to our manuscript, and we thank the editor for this suggestion.

5 - I may have missed it, but it will be useful to have a table of words usage, the source for the word-cloud images. I t can be a supplement table.

**Author’s response** – We now included a new Table (supplementary Table 2) in the manuscript with all the words and their frequencies used for the generation of the word clouds.

6 - Also, it will be very helpful to organize all the relevant factual numbers in one table: number of journals, number of articles in each one. Also add N=… to the figures or figure captions.

**Author’s response** – We now included a new Table in the manuscript containing all the study’s relevant factual numbers (number of journals, journal’s abbreviations, number of articles in each one) and we also added to all the figure captions the number of articles (N) used for each journal.

We hope that the modifications made in the new version of the manuscript have properly addressed the criticism and suggestions made by the referee, and that the improvements made in the manuscript will be sufficient for its publication in **PLOS ONE**.

I would like to state that all listed authors qualify for authorship and agreed in the submission of the manuscript. The final version of the manuscript has been seen and approved by all coauthors. The authors declare that they have no conflict of interest.

With kind regards,
